# Altered Breast Development in Young Girls from an Agricultural Environment

**DOI:** 10.1289/ehp.8280

**Published:** 2006-03

**Authors:** Elizabeth A. Guillette, Craig Conard, Fernando Lares, Maria Guadalupe Aguilar, John McLachlan, Louis J. Guillette

**Affiliations:** 1 Department of Anthropology, University of Florida, Gainesville, Florida, USA; 2 Center for Bioenvironmental Research, Tulane–Xavier Universities, New Orleans, Louisiana, USA; 3 Dirección del Area de Recursos Naturales, Instituto Tecnológico de Sonora, Ciudad Obregón, Sonora, Mexico; 4 Department of Zoology, University of Florida, Gainesville, Florida, USA

**Keywords:** breast development, mammary gland, Mexico, puberty, thelarche, Yaqui Valley

## Abstract

In several human populations, the age at which female breast development begins is reported to have declined over the last five decades. Much debate has occurred over whether this reported decline has actually occurred and what factors contribute to it. However, geographical patterns reflecting earlier developmental onset in some human populations suggest environmental factors influence this phenomenon. These factors include interactions between genetic makeup, nutrition, and possible cumulative exposure to estrogens, both endogenous as well as environmental beginning during *in utero* development. We examined the onset of breast development in a group of peripubertal girls from the Yaqui Valley of Sonora, Mexico. We observed that girls from valley towns, areas using modern agricultural practices, exhibited larger breast fields than those of girls living in the foothills who exhibited similar stature [e.g., weight, height, body mass index (BMI)], and genetic background. Further, girls from valley towns displayed a poorly defined relationship between breast size and mammary gland development, whereas girls from the Yaqui foothills, where traditional ranching occurs, show a robust positive relationship between breast size and mammary size. The differences noted were obtained by a medically based exam involving morphometric analysis and palpation of tissues, in contrast to visual staging alone. In fact, use of the Tanner scale, involving visual staging of breast development for puberty, detected no differences between the study populations. Mammary tissue, determined by palpation, was absent in 18.5% of the girls living in agricultural areas, although palpable breast adipose tissue was present. No relationship was seen between mammary diameter and weight or BMI in either population. These data suggest that future in-depth studies examining mammary tissue growth and fat deposition in breast tissue are required if we are to understand environmental influences on these phenomena.

The age for the onset of puberty is reported to have declined in many human populations over the last 50 years (Lee et al. 2001). Precocious puberty, in the United States, is now suggested to be initial breast and pubic hair development before 7 years of age for Caucasian-American girls and before 6 years of age for African-American girls (Kaplowitz and Oberfield 1999). The leading hypothesis for this change toward earlier puberty is better nutrition, with more rapid body growth, increased weight, and fat deposition. The rise in obesity is also considered a contributing factor (Styne 2004; Wattigney et al. 1999). The amount of fat tissue in the body, as early as 5 years of age, is reported to be correlated with earlier puberty in Caucasians but less so in African Americans (Davison et al. 2003). Such findings imply that the lower age of female breast development, in light of nutritional change, is a normal phenomenon, with a proposal of revised norms. The question arises, “How early can breast development occur and still be normal?” Basic morphometrics and descriptive gross anatomy [i.e., standard Tanner scale (Tanner 1962) for puberty landmarks of breast and pubic hair development] remain the standard data collected to access the initiation and staging of puberty in many clinical settings (Sun et al. 2002). The Tanner scale (Tanner 1962), which involves the use of pictures of the breast reflecting developmental stages from the absence of development (stage 1) to adult breast development (stage 5), is based mainly on external morphology. Current norms are based on this visual scaling. The type of tissue growth within the breast itself, including mammary gland development, is poorly investigated in the human female, with the assumption that initial breast growth in girls is tightly correlated with the development of mammary tissue and a variable deposition of fat tissue. We addressed this assumption when girls from reference and agricultural, chemically exposed populations were examined. We hypothesize that an altered relationship between breast size, fat deposition, and mammary tissue development could result from *in utero* and/or childhood exposures to estrogenic or antiandrogenic chemicals, as has been reported in studies of laboratory rodents (Brown and Lamartiniere 1995; Vorderstrasse et al. 2004). At present, we know of no human data directly examining contaminant exposure and initial development of the pubescent mammary gland. Several studies have examined contaminants and the prevalence of precocious puberty (for review, see Parent et al. 2003).

In 1969, initial breast development was reported to occur at 11 years of age, on average, in English girls, with fuller breast development and the appearance of pubic hair and menarche expected by age 13 (Marshall and Tanner 1969). By 1997, of the 17,077 girls seen in over 200 pediatricians’ offices, 14% of the American-Caucasian girls and 48% of the African-American girls were reported to have breast development by 8 years of age (Herman-Giddens et al. 1997). Mexican-American girls developed later than African-American girls, but any significant difference between Mexican-American girls and Caucasians remains questionable (Sun et al. 2002; Wu et al. 2002). Organochlorine pesticides are believed to be responsible for precocious puberty that occurred in girls immigrating at very young ages from countries using dichlorodiphenyltrichloroethane (DDT); breast development occurred at 8 years of age and menarche at 10 years of age in these girls. High blood levels of DDT were present in 21 of the 26 children. Genetic factors were not believed to be involved (Krstevska-Konstantinova 2001).

The process and timing of puberty is influenced by complex interactions between neural and sex hormones. Breast development is a component of puberty, occurring in the continuum of the development of gonadal function and the ontogeny of the hypothalamic–pituitary–gonadal axis, beginning in the fetus and continuing until adulthood (Grumbach 2002). The role of endocrine-disrupting chemicals (EDCs) on the puberty continuum has received limited attention, but several reviews suggest a need for more research (Bern 1992; Nebesio and Pescovitz 2005; Parent et al. 2003; Wang et al. 2005). The exposure of laboratory animals and wildlife to EDCs is known to alter the ratio of female to male hormones that play a dominant role in sexual development (Gray et al. 2002; Guillette and Gunderson 2001; McLachlan 2001). Exposure to some estrogen mimics or antiandrogens can delay puberty in female rodents (Monosson et al. 1999), whereas experimental exposure to low doses of estrogenic bisphenol A, found in some plastics, speeds growth and puberty in rats (Howdeshell et al. 1999).

Our ability to detect the possible role of xenobiotic chemicals in altering pubertal development is confounded in modern societies by the many nutritional, genetic, and lifestyle factors capable of affecting puberty. The Yaqui Valley of Sonora, Mexico, provides an ideal setting for a study examining the potential role of agriculturally derived contaminants in promoting premature or abnormal breast development during puberty. The Native-American inhabitants of this valley split philosophically over the use of modern agriculture, including pesticide application, during the Green Revolution of the early 1950s. The result was a geographical division of towns, including a division of extended families, based on a continuation of traditional ranching and home gardens versus the use of newly introduced agricultural methods (Almada-Bay 2000). The children of three small agriculturally based towns located in the Yaqui Valley were examined; the fields surrounding the towns have monocropped fields with pesticide applied aerially. In contrast, the reference site is located 80 km northwest in the Sierra Madre Mountain foothills. Income in this town is from cattle ranching, with few homes having home gardens. The initial refusal to use pesticides continues. The towns used in this study are those included in previous research studies on childhood growth and development where food sources and nutritional, economic, and social status were initially investigated and found to have changed little over recent years (Guillette 2003; Guillette et al. 1998). All towns have similar modernization, infrastructure, and socioeconomic conditions, with > 90% living in poverty, limiting general EDC exposures. Hard plastic plates are used by all; the use of plastic for shopping or other activities in the kitchen and home are absent. Cement flooring and wood furniture limit exposure from chemical offgassing. Women traditionally do not wear makeup or use artificial scents either on the body or in the home. Approximately half the homes at all study sites have television (determined by counting television aerials). Both the modern agriculturally based towns as well as the cattle-producing foothill towns using no pesticides maintain traditional customs and similar parenting practices. There is a continuation of intertribal marriage patterns that now reflect an individual’s philosophical stance over the use of modern farming practices. Past and present dietary studies have determined that the main source of food originates from the capital city, with types of food and the amount served similar in the two areas. Further, women in these communities report no tobacco or alcohol use, reflective of traditional mores. Usage among men is minimal (Guillette et al. 1998). The populations of these towns (800–1,000 people) limited the number of children available in each age group. In a previous study (data collected in 1996), the children 4 to 5 years of age in each of the three towns where agricultural pesticide use occurred exhibited the same multiple developmental task delays and neuromuscular and mental deficits compared with children living in the reference town (Guillette et al. 1998). Developmental tasks and problem solving continued to be delayed with the same groups of exposed children 2 years later (Guillette 2003).

Cord blood studies in 1990, from infants born in these agricultural towns 2 years before the birth of the girls in the current study, indicated transplacental transfer of relatively high levels of various organochlorines, including lindane, heptachlor, aldrin, dieldrin, endrin, and DDT metabolites (Garcia and Meza 1991). An investigation of dumping areas for pesticide containers included such containers in 1996, with pyrethroids and carbonates added in a 1999 inspection, and malathion compounds noted in 2001, after which such sites were banned.

Agricultural activity has sharply declined in the valley since 2000 because of a long-term drought. At the time of the current study (2003), drought had persisted for 5 years. Planted fields were difficult to find, as an overwhelming reversal to ranching had occurred. Hence, present-day acute exposure to pesticides by dermal absorption and by inhalation has been greatly reduced in the valley. Because pesticides are ubiquitous in the environment and the reference town had annual spraying by the government for malaria control until 2000, the populations of children at the reference site have been considered less exposed. Both groups continue to be exposed through ingestion of pesticide residues on purchased foods.

## Methods

For this research we examined a group of Native American (Sonora Mayan) girls, each with parents who have resided in the town since birth. This study examined thelarche, the onset of puberty in terms of breast development, in female children 8–10 years of age. Human study permission was granted from Tulane University and the State of Sonora Institutional Review Board (IRB). The Medical Council of Sonora IRB ruled that *a*) all the girls must be known by the local nurses and *b*) all had to have had previous examinations by the town health clinic nurse or physician. The selection of the girls by the clinical staff prohibited a true random selection, although the number of girls in the age group of interest was limited because of the small size of the towns. The time involved to obtain IRB permission from Mexico eliminated the involvement of the oldest girls examined during the previous studies of children from this region. A few of the previously studied females, however, were included.

A total of 50 girls were examined, 30 from the three agriculturally located towns and 20 from the nonagriculture town. All were classified as healthy children without birth defects or tumors. The local clinic health provider plus the principal investigator (E.A.G.), also medically trained, examined each child in terms of growth, in the presence of the child’s mother. Evaluation of breast development was determined by these two individuals and involved visual assessment using the Tanner scale as well as morphometric data. The Tanner scale, pictorially demonstrating five stages of breast growth, was used as one measurement of pubertal stage. For Tanner scaling, independent ratings were made by the principal investigator and the clinic health provider. Measurements obtained included height, weight, sitting height, chest circumference at the bust line, chest circumference 3 cm below the bust line, and waist and hip circumferences. A standard fat fold measurement from the right arm triceps was also taken. Measurements were made by palpation of the diameter of firm, internal, mammary tissue as well as the diameter of the softer, external breast field. The right breast diameter, determined by adipose deposits, was delineated by palpation and measured in centimeters. The girls resisted the use of calibers, fearing pain, so the diameter was determined by placing the index finger of each hand vertically on each side of the breast, with the distance between measured by the second person, using a measuring tape. The breast was then palpated for mammary tissue, which is firm compared with the softer fatty breast tissue. The diameter of the mammary tissue was measured in the same manner described above. Each girl was measured twice, with a reversal of roles by the clinic health provider and lead investigator. If disagreement was more than 0.5 cm, new measurements were taken until consensus was reached by both researchers.

Statistical analyses were performed on all parametric data using analysis of variance (ANOVA) or analysis of covariance (ANCOVA) with post hoc analysis using the software StatView 5.0 (SAS Institute Inc., Cary, North Carolina, USA). Initially, the four towns were analyzed as separate groups, even though samples size was approximately 10 for each agricultural town. No differences were noted among the three valley towns, and they were grouped, thus increasing the power of the statistical analyses. For measurements where two independent values were obtained (i.e., Tanner stage), a mean was calculated for each girl and used in further analyses. Homoscedasticity of variance among samples was tested using an *F* test. If variance was heterogeneous, data were log transformed to achieve homogeneity. Simple, stepwise, and multiple regression analyses were performed to examine the possible influence of the various independent variables on the dependent variables (mammary and breast diameter). These analyses were followed by ANOVA or ANCOVA, as appropriate. For all statistical tests, *p* < 0.05 was considered significant.

## Results

Background data obtained from the mothers indicated that diet and types of daily activity, including play and television, were similar for girls from the four study towns. Data were initially analyzed by town. We observed that girls from the three agricultural and one reference sites had no significant differences in age, height, weight, or other body measurements; thus, we combined the agricultural sites and present the data for two regions, the valley with greater pesticide exposure and the foothills with less exposure (Table 1). Importantly, we noted that the children in our study did not differ significantly in body mass index (BMI), based on age group, from standardized averages of BMI reported for Hispanic children by Rosner et al. (1998). We did note that variance was heterogeneous for a number of variables within the exposed valley population exhibiting significantly elevated variance relative to the reference population in the foothills. Head circumference (*F* = 24.2, *p* < 0.0001) exhibited a highly significant variation in the population of exposed girls as did weight (*F* = 3.85; *p* = 0.002), BMI (*F* = 3.72; *p* = 0.003), and upper arm circumference (*F* = 2.39; *p* = 0.04).

We made two independent assessments of Tanner stage and measurements for mammary and breast diameters. No difference in Tanner stage for breast development was observed (*p* = 0.4) among girls from the two regions, as girls from the valley averaged (mean ± SE) 2.4 ± 0.2 on the Tanner scale for breast development whereas girls from the foothills averaged 2.15 ± 0.2. In contrast to the analysis by Tanner stage, morphometric analyses demonstrated that breast development was different among sites. Girls were staged as prepubescent if no mammary budding could be observed or palpated in the breast field. Using the traditional Tanner scale of visual staging, 6 of 20 (30%) less-exposed girls for the foothills and 6 of 30 (20%) girls from the more-exposed valley towns visually exhibited Tanner stage 1—prepubertal with no budding of the nipple or fat deposits. Using palpation, however, three of the six girls from the valley population lacked fatty deposits. Among the exposed girls from the valley towns exhibiting breast development, mammary tissue could not be palpated in 5 of the remaining 27 pubescent girls. These girls without palpable mammary tissue had breast diameters ranging from 3.0 to 12.0 cm (8.1 ± 1.6 cm). The 12 girls lacking palpable mammary tissue were not significantly different in height (*p* = 0.79) or weight (*p* = 0.20) when compared with girls with mammary tissue; thus, the girls lacking mammary development do not represent the youngest, heaviest, or smallest girls. None of the pubescent, less-exposed girls from the foothills exhibiting breast development (Tanner scores 2 or greater) lacked palpable mammary tissue.

**Figure 1:**
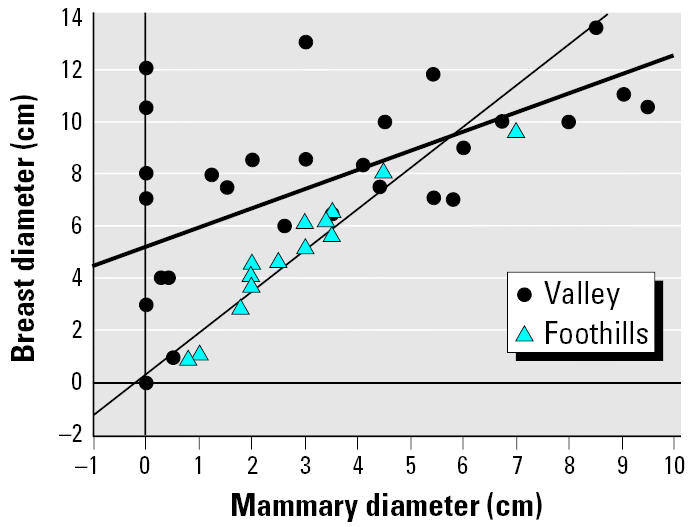
Relationship between mammary diameter and breast diameter in peripubescent girls from two populations in the Yaqui Valley, Mexico.

**Figure 2:**
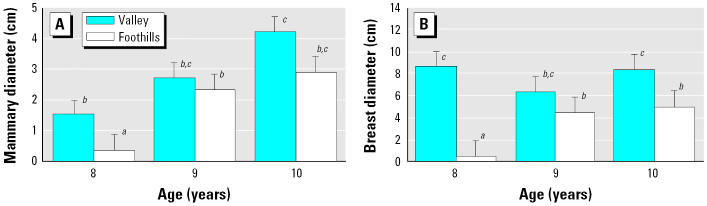
Mean (± SE) mammary (*A*) and breast (*B*) diameter in peripubescent girls 8–10 years of age from two populations in the Yaqui Valley, Mexico. ***^a–d^***Bars having different superscripts indicate a significant difference.

Breast diameter was greatly influenced by mammary diameter in the less-exposed girls from the foothills, with 90% of the variation in breast diameter explained by mammary diameter (Figure 1). This relationship was much weaker in girls from the valley, with only 27% of breast diameter explained by mammary tissue diameter (Figure 1). Mammary size was not different between the regions (*p* = 0.65) based on an ANOVA, but age did influence mammary size (*F* = 4.9; *p* = 0.01); the interaction of region and age on mammary size was not significant (*p* = 0.88) (Figure 2A). As mammary size correlates with and directly influences breast size, an ANCOVA was performed to determine possible differences in breast size among age groups and regions, with mammary size as a covariate. Breast size was significantly different between regions (*F* = 30.9; *p* < 0.0001) and age groups (*F* = 3.15; *p* = 0.05); the interaction between these variables was also highly significant (*F* = 26.9; *p* < 0.0001). Breast diameter at 8 and 10 years of age was significantly larger in the exposed valley girls when compared with girls from the less-exposed foothills (Figure 2B).

Breast diameter was weakly correlated with body weight and height in the exposed girls from the valley but not in the less-exposed girls from the foothills (Table 2). No relationship was seen between BMI and breast diameter in either population. Mammary diameter showed a relationship with body height in the exposed girls but no relationship with those from the foothills (Table 2). No relationship was seen between mammary diameter and weight or BMI in either population. Multiple regression analysis using three variables (mammary diameter, body weight, and body height), shown to have a relationship with breast size in at least one of the populations (valley), was performed to examine the relationship with breast size. In the valley population, 54.2% of the variation in breast size was explained by mammary diameter and individual body weight, with no additional clarification by the inclusion of height. In the foothill population, 91.7% of the variation in breast diameter in the girls was explained by mammary size alone, and weight and height did not add further resolution in explaining mammary diameter.

## Discussion

In two groups of girls, differing primarily in the degree of exposure to agricultural chemicals, we observed the following: *a*) Visual categorization of breast development did not predict mammary development in girls with elevated contaminant exposure, suggesting that this visual technique, used in most cross-sectional and longitudinal puberty timing studies published over the past 50 years that assessed breast development stage, may have missed critical differences. In fact, a number of girls with apparent breast development had no palpable mammary tissue. *b*) Fat deposition in the breast appears to follow a different pattern in girls living in an environment with elevated contaminant exposure when compared with a population of girls with less exposure. Interestingly, female fat deposition patterns for the agricultural sites, such as in the hips, were not different. These data suggest that the onset or pattern of thelarche in girls living in the agricultural areas may be altered from that of girls exposed to lower levels of agricultural chemicals. *c*) Few differences were noted in average body measurements other than breast diameter, yet we noted significant differences in the variance of weight and upper arm and head circumference in the valley population indicative of affected individuals. Variance needs to be used to assess responses as well as changes in the population’s central tendency, such as the means in mammary diameter and head circumference. Altered variance is a positive indicator of exposure and effect (Orlando and Guillette 2001). Given the complex mixture of pesticide exposure for the mothers and daughters in the agricultural area, and the variation in both dose and timing of exposure, the resulting heterogeneity of variance is a likely indicator of an exposure effect (Orlando and Guillette 2001). If head circumference is one measure of brain growth, then the highly significant variance in this morphological measurement—similar to previous findings reported for boys and girls together (Orlando and Guillette 2001)—suggest that further study should be undertaken to access the neurological deficits found previously in children from this population (Guillette et al. 1998).

Using a morphometric approach, we observed that girls living in the valley exhibited a different pattern of breast development when compared with girls of similar age and body size living in the foothills. Yet if this comparison were based on the traditional based Tanner scale, no differences would have been noted. There was a very different relationship between breast development and a girl’s weight and height if that girl came from the valley, where contamination is higher, rather than the foothill population where environmental contaminant exposure is presumably lower. Breast development and size in girls from the foothills was explained almost exclusively by growth in mammary tissue, whereas breast development in girls from the valley was less defined by mammary tissue growth. Further, the lack of differences between the two groups when measurements of bust, lower chest (3 cm below the bust), waist, and hip were compared suggests that the exposed girls, as a group, were not greatly advanced in other aspects of pubertal development involving overall female fat deposition.

Pubertal breast development, including fat deposition, occurs through an increase in circulating concentrations of sex steroids as well as increasing tissue concentrations of estrogens (primarily estradiol-17β) (Boyd et al. 1996). Unfortunately, we were unable to obtain IRB permission to obtain blood samples from the girls in this study. Mammary gland development at puberty is dependent upon normal mammary gland differentiation in the embryo and elevated estrogen concentrations during puberty. Boyd et al. (1996) examined estrogen receptor alpha (ER-α) mRNA expression and its protein product in tissue samples from 89 breasts from clinically normal female infants, children, adolescents, and adult pre-menopausal and postmenopausal women. They noted that mRNA expression of the ER-αgene varied with hormonal status as expected based on laboratory studies.

We noted in this study that breast diameter was weakly correlated with body weight and height in the exposed girls from the valley but not in the less-exposed girls from the foothills. No relationship was seen between BMI and breast diameter in either population. Mammary diameter showed a relationship with body height in the exposed girls but no relationship with those from the foothills. No relationship was seen between mammary diameter and weight or BMI in either population. Our observations are in contrast to at least one study showing that a BMI was predictive of earlier onset of puberty in girls (Kaplowitz et al. 2001). Instead of using a raw BMI score, Kaplowitz et al. (2001) used a normalized score based on standardized age-specific average BMI scores produced from a large study of children of various ethnic backgrounds (Rosner et al. 1998). We performed a similar analysis (not presented here) using normalized BMI scores, but still observed no relationship between BMI and breast or mammary diameter. Importantly, we noted that the children in our study did not differ significantly in BMI, based on age group, from the averages reported by Rosner et al. (1998) for Hispanic children. Thus, our lack of a relationship between BMI and breast development is unlikely to be due to the fact that the children studied here are significantly different in weight and height compared with other populations of children of similar age.

Exposure during critical windows would affect mammary development. We proposed the hypothesis that the changes reported here in the peripubertal girls from the Yaqui Valley of Mexico are due to *in utero* exposure to agricultural chemicals with endocrine action. This study was designed to test this hypothesis, as a principal difference between the two populations studied was parental exposure to agricultural chemicals. Various pesticides, mainly organophosphates and organochlorines, were used extensively in the agricultural areas of the Yaqui Valley near the time of the girls’ birth (1992–1994), and many of these compounds are known to cross the placenta. A study of newborn children from the Yaqui Valley performed close to the period these children were conceived reported elevated pesticide levels, with cord blood values of lindane, heptachlor, benzene hexachloride, aldrin, and endrin all exceeding World Health Organization established limits (International Programme on Chemical Safety 2005), and *p,p*′-dichlorodiphenyldichloroethylene (*p,p*′-DDE) measured in the parts per million (Garcia and Meza 1991). Both lindane and *p,p*′-DDE are estrogenic in the MCF-7 mammary tumor cell line (Soto and Sonnenschein 2000). It is important to note that this geographic region was in the fifth year of a drought at the time of this study, with most farmers moving into ranching, consequently decreasing daily exposure to pesticides. Thus, our observations are likely related to embryonic, neonatal, or early childhood exposure and are unlikely to be the result of elevated exposure during puberty.

Human exposure to EDCs, either prenatally or during childhood, should be considered a plausible factor altering breast development and the timing of puberty. In girls exposed through breast feeding to polybrominated biphenyls, observable pubic hair occurred earlier than less-exposed girls, but little association was found with breast development (Blanck et al. 2000). High levels of DDT metabolites have been found with girls experiencing early secondary sex characteristics (Bongiovanni 1983). Mice exposed during embryonic development to the environmental toxicant dioxin exhibit disrupted mammary gland differentiation that includes stunted growth, decreased branching, and poor formation of lobular alveolar structures (Fenton et. al. 2002; Vorderstrasse et al. 2004). Together, these data suggest that future studies should include an in-depth examination of mammary tissue growth and fat deposition in a girl’s breast, not Tanner staging alone. Palpation, differentiating between fat and mammary tissue, provides only an indication of mammary development. The small, less-developed rural towns and lack of available sophisticated equipment found in more technological advanced areas limited the depth of this study. Future studies, both longitudinal and cross-sectional, using measurement techniques acceptable to the child or technological equipment providing visualization of tissues, need to be done to evaluate types and amount of tissue growth and abnormal correlations between the two types of tissue. Exposure to varied environmental factors, including nutrition and contamination, is ubiquitous; thus, all studies examining thelarche must examine nutrition and contamination histories as well as the age of onset and ethnicity.

Mammary tissue maturation, necessary for breast-feeding, is an important question that remains for these girls and requires further investigation. The shortened period of lactation found by Gladen and Rogan (1995) in Mexican agriculture-based mothers may be reflective of poor mammary structure and not solely to blood levels of DDE or other pesticides at the time of infant delivery. Any adverse alterations in lobular and duct formation could lead to alterations in breast maturation during pregnancy followed by adverse effects on lactation. Such alterations would have great implications for populations that live in poverty, where breast feeding is a necessity for neonatal survival and health. In addition, the question remains whether these alterations influence the incidence of disease later in life, such as obesity or breast cancer. It is critical that mammary gland growth and development be examined in far more detail in future studies of puberty in females.

## Figures and Tables

**Table 1:** Values (mean ± SE) for various morphological features measured on young girls from the valley and foothills populations in the Yaqui Valley, Mexico.

Measurement	Valley (*n* = 30)	Foothill (*n* = 20)	*F*-value (*p*-value)
Weight (kg)	38.6 ± 2.55	33.83 ± 1.59	1.98 (0.17)
Height (cm)	137.1 ± 1.49	137.0 ± 2.27	0.002 (0.96)
BMI	20.6 ± 1.4	18.1 ± 0.9	1.76 (0.19)
Sit height (cm)	70.1 ± 0.9	70.4 ± 1.03	0.05 (0.8)
Chest circumference (cm)	68.8 ± 1.6	68.5 ± 1.8	0.02 (0.9)
Chest circumference–3 cm (cm)	64.5 ± 1.4	63.9 ± 1.7	0.07 (0.8)
Waist circumference (cm)	63.1 ± 1.5	63.3 ± 1.8	0.009 (0.9)
Hip circumference (cm)	76.8 ± 1.6	76.4 ± 1.7	0.04 (0.9)
Upper arm circumference (cm)	21.0 ± 0.66	21.3 ± 0.5	0.12 (0.7)
Upper arm fold (mm)	19.2 ± 1.5	21.75 ± 1.4	1.4 (0.2)
Head circumference (cm)	52.8 ± 1.45	53.8 ± 0.4	0.3 (0.6)
Age (years)	9.2 ± 0.13	9.2 ± 0.19	0.095 (0.8)

**Table 2:** Simple and multiple regression analyses examining the relationships between mammary or breast diameter and height, weight, or BMI in girls from two populations in the Yaqui Valley, Mexico.

	Valley		Foothills		
	*F*-value (*p*-Value)	*R*^2^	*F*-value (*p*-Value)	*R*^2^
Mammary diameter versus				
Height	13.3 (0.0012)	0.35	0.113 (0.74)	0.009
Weight	0.41 (0.53)	0.02	2.74 (0.12)	0.17
BMI	2.87 (0.10)	0.10	3.11 (0.10)	0.19
Breast diameter versus				
Height	10.9 (0.0029)	0.30	0.72 (0.41)	0.05
Weight	5.02 (0.034)	0.17	4.18 (0.062)	0.24
BMI	1.09 (0.31)	0.04	2.8 (0.115)	0.18
Multiple				
Mammary diameter, weight, and height	9.08 (0.0004)	0.54	40.67 (< 0.0001)	0.917
